# Relevance of a subjective quality of life questionnaire for long-term homeless persons with schizophrenia

**DOI:** 10.1186/s12888-017-1227-0

**Published:** 2017-02-17

**Authors:** V. Girard, A. Tinland, J. P. Bonin, F. Olive, J. Poule, C. Lancon, T. Apostolidis, M. Rowe, T. Greacen, M. C. Simeoni

**Affiliations:** 1 Public Health Research Unit EA 3279, Aix-Marseille University, Marseille, France; 2 Community Mental Health Outreach Team, MARS (Movement and Action for Social Recovery), Public Hospital of Marseille (AP-HM), Marseille, France; 3 School of Nursing, University of Montreal and Fernand-Seguin Research Centre, Québec, Canada; 4 Social Psychology Unit EA849, Aix-Marseille University, 13621 Aix-en-Provence, France; 5 Yale Program for Recovery and Community Health, Yale University, New Haven, USA; 6 Maison Blanche Hospital Research Laboratory, Paris, France; 7 Aix Marseille Université, LPS EA 849, 13621 Aix en Provence, France; 8 Service d’évaluation médicale, AP-HM, Conception, 13005 Marseille, France

**Keywords:** Subjective measure, Validation, Schizophrenia, Homeless, Extreme inequalities

## Abstract

**Background:**

Increasing numbers of programs are addressing the specific needs of homeless people with schizophrenia in terms of access to housing, healthcare, basic human rights and other domains. Although quality of life scales are being used to evaluate such programs, few instruments have been validated for people with schizophrenia and none for people with schizophrenia who experience major social problems such as homelessness. The aim of the present study was to validate the French version of the S-QoL a self-administered, subjective quality of life questionnaire specific to schizophrenia for people with schizophrenia who are homeless.

**Methods:**

In a two-step process, the S-QoL was first administered to two independent convenience samples of long-term homeless people with schizophrenia in Marseille, France. The objective of the first step was to analyse the psychometric properties of the S-QoL. The objective of the second step was to examine, through qualitative interviews with members of the population in question, the relevance and acceptability of the principle quality of life indicators used in the S-QoL instrument.

**Results:**

Although the psychometric characteristics of the S-QoL were found to be globally satisfactory, from the point of view of the people being interviewed, acceptability was poor. Respondents frequently interrupted participation complaining that questionnaire items did not take into account the specific context of life on the streets.

**Conclusions:**

Less intrusive questions, more readily understandable vocabulary and greater relevance to subjects’ living conditions are needed to improve the S-QoL questionnaire for this population. A modular questionnaire with context specific sections or specific quality of life instruments for socially excluded populations may well be the way forward.

**Electronic supplementary material:**

The online version of this article (doi:10.1186/s12888-017-1227-0) contains supplementary material, which is available to authorized users.

## Background

Homelessness among people living with schizophrenia is increasingly visible on the streets of major cities in developed countries [1]. In 2011, the European Community addressed the problem of long-term homelessness^1^ and its impact on those with severe psychiatric disorders [2]. This group has a high mortality rate [3] that is associated with length of time spent living on the streets [4].

New interventions have been developed over the past thirty years to address this issue, with significant positive impacts on the living conditions and the mental and physical health of the populations in question [5]. Quality of life is regarded as a significant criterion in evaluating the efficacy of these interventions [6]. Instruments currently used to assess quality of life in this population are either generic scales validated for the general population [7] or specific scales for people living with schizophrenia [8].

The only quality of life scale validated for homeless people with severe psychiatric disorders is the Lehman Quality of Life Interview (QOLI) [9]. This scale, however, has not been validated for use with homeless people with schizophrenia. In addition, it was constructed from the point of view of experts and clinicians, not of the patients themselves. Scales constructed from the point of view of experts have two weaknesses. First, they are seldom correlated with scales of clinical symptoms [10]. Second, discrepancies between clinicians’ and patients’ evaluation of the latter group’s quality of life are common [11].

In order to better take into account the lived experience of schizophrenia, researchers have constructed scales starting from the point of view of the people who experience it [12]. One such scale is the S-QoL, or Schizophrenia Quality of Life Scale [13]. The S-QoL has greater sensitivity to clinical change than generic quality of life scales, and has been shown to be well correlated with patients’ clinical symptoms [13]. It also has the advantage of being short (15 minutes) and self-administered [13].

Despite the advantages of scales such as the S-QoL, methodological problems inevitably arise when structured questionnaires designed for the general population are used in homeless populations [14], as homelessness may significantly alter the subjective experience of persons with schizophrenia [15]. Risk factors associated with homelessness for people with schizophrenia who are not homeless suggest that their life experiences and priorities are highly likely to differ from those who are. These include, for the latter group, coming from a dysfunctional family, illness severity, less frequent use of social services [16], ethnicity (with persons of African origin being at higher risk) and addictive behaviour [17]. Consequently, some aspects of quality of life evaluated by the S-QoL for the general population may be less relevant for people who are homeless and may fail to capture certain aspects of their quality of life, such as those related to extreme disparities in access to health [18].

Overall, the fact that the S-QoL may not cover all the domains of quality of life relevant for people who are homeless challenges the content validity of this questionnaire for this group.

Furthermore, self-report, as recommended for the S-QoL questionnaire, may be inappropriate for homeless persons, since comprehension difficulties are frequent encountered owing to both illness severity and high rates of substance use. The aim of the present study was to assess the reliability, the content and construct validity, and the acceptability of the S-QoL in long-term homeless adults living with schizophrenia.

## Methods

The study population was homeless adults living with schizophrenia who were patients of an outreach team in Marseille, a major city in the south of France. All participants were long-term homeless, defined as being continuously homeless for at least one year or having had four episodes of homelessness in the past three years. Only patients who spoke French were eligible to participate. The team psychiatrist confirmed the diagnosis of schizophrenia, based on DSM IV-TR classification criteria. Those meeting the inclusion criteria were approached by a trained social psychologist, on the street or at other sites, to obtain their oral informed consent to participate in the study. The psychologist and the outreach were in charge to evaluate together if the patient was well enough to consent for himself.

### The study took place in two successive steps

The aim of the first step was to analyse the psychometric characteristic of the S-Qol questionnaire in a convenience sample of 55 adult patients diagnosed with schizophrenia and who agreed to complete a research questionnaire including the S-QoL instrument.

The second step explored the acceptability and content validity of the S-QoL for this population. Twenty-one additional subjects with the same enrolment criteria as the fist group were recruited 12 months following completion of the first step, and with no duplication of subjects. Subjects participated in individual interviews on the S-QoL instrument, including a cognitive debriefing.

Data collection took place from August 2010 to May 2014. The study was conducted in compliance with the Declaration of Helsinki and in accordance with good clinical practice [19].

In the first step, socio-demographic data and the Clinical Global Impression (CGI) and S-QoL scales were collected for all patients. The outreach team psychiatrist who made the diagnosis of schizophrenia completed the CGI evaluating mental illness severity [20] on a scale from 1 (not severe at all) to 7 (among the most severe). A social psychologist trained in participant observation research [21] then met with prospective participants several times before conducting the research interview. During the research interview, she first collected socio demographic data and then asked the subject to complete the S-QoL questionnaire.

The researcher took notes on participants’ remarks while they were answering or filling in the questionnaire and immediately following completion.

In the second part of the study, qualitative interviews were conducted based on the *pretesting technique* [22]: subjects were invited to read each question in the S-QoL questionnaire out loud and to comment on each. They were encouraged to express themselves as spontaneously as possible, saying *whatever came into their minds*. The investigator collected the subjects’ responses to each item of the questionnaire and took notes concerning their intellectual and emotional reactions, as she perceived them. Following completion of commentary on all items, subjects were asked six open-ended questions:

- 1. What did you think of the questionnaire?
- 2. Did you have any trouble understanding it?
- 3. What did you think of how the questions were worded, such as how long or short they were?
- 4. Were there any questions that seemed to you to be inappropriate?
- 5. Was anything left out of this questionnaire?
- 6. What is *quality of life,* as you see it?

*Schizophrenia Quality of Life Questionnaire (S-QoL)* [13]: This health-related quality of life questionnaire was developed from the point of view of persons living with schizophrenia. The questionnaire includes 41 items covering eight dimensions: psychological well-being (PsW), self-esteem (SE), relations with the family (RFa), relations with friends (Rfr), resilience (RE), physical well-being (PhW), autonomy (AU) and sentimental life (SL). Each item is evaluated using a 5-point Likert scale from 1 (less than desired) to 5 (more than desired). The scores for each dimension and the index vary from 0 (low quality of life) to 100 (high quality of life).

#### Step 1: Analysis of the psychometric properties of the S-QoL instrument in a sample of 55 long-term homeless persons with schizophrenia

Analysis of the distribution of dimension scores of the S-QoL was carried out to identify eventual floor and ceiling effects. Internal consistency reliability was studied using the Cronbach alpha coefficient. An alpha value greater than 0.7 was considered to be satisfactory (Table 1).

**Table 1:** Characteristics of participants in two steps of the long term homeless study (), (*n* = 55)

	Characteristics	Step 1 *N* = 55 N (%)	Step 2 *N* = 21 N (%)
Gender	Male	40 (72.7)	18 (85.7)
Age, y	<30	4 (7.3)	2 (9.5)
	31 – 40	17 (30.9)	8 (38.0)
	41 – 50	17 (30.9)	7 (33.3)
	>50	17 (30.9)	4 (19.0)
Marital status	Single	39 (70.0)	19 (90.5)
Education	High school or more	13 (23.6)	4 (19.0)
Type of schizophrenia	Paranoïd	49 (89.1)	15 (71.0)
Severity of illness, CGI score	1–3	2 (3.6)	1 (4.7)
	4–5	19 (34.6)	7 (33.3)
	6–7	34 (61.8)	13 (61.9)
Pharmacological treatment	Yes	47 (87.5)	20 (95.2)

CGI, Clinical Global Impression

Assessment of construct validity was twofold:

1. The Rasch Rating Scale Model (RSM), which is a part of Item Response Theory (IRT), was used to explore the uni-dimensionality of each domain [23]. Uni-dimensionality was retained for INFIT index values ranging from 0.7 to 1.3.
2. Item-dimension score correlations were computed using Spearman’s correlation coefficients. The construct validity was considered satisfactory for a correlation of each item with its dimension corrected for overlap (item internal consistency, IIC) both greater than 0.4 and greater than the correlation of this item with the other dimensions (item discriminant validity, IDV).

SPSS 17.0, MAP, and Winstep software were employed.

#### Step 2: Subjects’ assessments of the S-QoL questionnaire

Thematic analysis was performed by three researchers from subjects’ responses and the researcher’s notes [24].

Our manuscript follow the STROBE guidelines for the reporting of observational studies.

## Results

### Step 1: Psychometric properties of the S-QoL

In the first part of the study, of the 98 persons in the outreach team's active patient list in 2010, 75 met with the investigator and 55 agreed to complete the questionnaire; 40 were men and 15 were women; Almost 90% of these 55 participants (*n* = 49) had a diagnosis of paranoid schizophrenia. With regard to participants' clinical severity, the CGI varied from 3 (mild severity) to 7 (among the most severe), with almost two out of three subjects (*n* = 34) with a score > 6. In the second part of the study, the 21 persons interviewed included 18 men and 3 women, with an average age of 42 (range 27 – 62).

Although the S-QoL questionnaire was completed in its entirety for all 55 participants, no single patient completed it entirely on their own. Although the S-QoL has been validated as a self-administered questionnaire, the first subjects interviewed were proved unable to complete the questionnaire on their own. It was therefore decided to resort to interviewer-administration.

The psychometric properties of the S-QoL questionnaire for the 55 long-term homeless persons with schizophrenia interviewed in the present study are displayed in Table 2.

**Table 2:** Reliability and construct validity of the SQoL in the homeless sample (MARS)

Dimension/Index (Number of items)	Mean (SD)	Item internal consistency min-max	Item discriminant validity min-max	Floor % (%ini^a^)	Ceiling % (%ini^a^)	Alpha	Infit min-max
PsW^b^ (10)	59.2 (26.3)	0.41–0.64	−0.04-0.66	19.8 (2.1)	41.6 (6.3)	0.86	0.75-1.32
SE^b^ (6)	55.2 (20.7)	0.42–0.70	−0.15–0.68	13.5 (2.5)	4.2 (4.5)	0.80	0.73–1.24
RFa^b^ (5)	38.4 (28.4)	0.75–0.87	−0.13–0.64	30.9 (6.3)	1.1 (4.8)	0.92	0.65–1.43
RFr^b^ (5)	53.3 (23.1)	0.61–0.74	−0.11–0.35	12.3 (5.9)	3.2 (3.7)	0.86	0.75–1.11
RE^b^ (5)	49 (19.9)	0.39–0.49	−0.12–0.51	12.7 (3.6)	14.7 (1.6)	0.70	0.93–1.17
PhW^b^ (4)	45.7 (22.8)	0.36–0.68	−0.09–0.49	18.2 (7.0)	2.7 (3.5)	0.77	0.72–1.36
AU^b^ (4)	61 (19.3)	0.14–0.57	−0.20–0.48	12.2 (4.9)	4.9 (4.4)	0.64	0.62–1.49
SL^b^ (2)	40.9 (26.7)	0.37	−0.06–0.38	29.1 (18.7)	1.8 (8.8)	0.54	0.88–1.10
Index^b^ (41)	50.3 (14.1)	NA	NA	NA	NA	0.79	NA

expliciter les noms des dimensions

^a^% ini : Floor/Ceiling effects on dimension scores in the not homeless reference population (SQoL-41 validation) ^b^theorical min = 0 and theorical max = 100

Floor and ceiling effects greater than 20% were found for three different dimensions of the S-QoL scale, whereas in the validation study, floor and ceiling effects were systematically below 10%.

Internal consistency reliability was satisfactory for the Index and for all dimensions with the exception of *Sentimental Life* (SL) and *Autonomy* (AU). Cronbach’s alpha for the AU dimension could be improved by deleting item 13 *'I can go out (to the cinema, for a walk, to a restaurant etc.)'* (alpha-value, if deleted, is 0.73), which weakly correlated to the total AU score (*r* = 0.14).

A total of 29 of the 41 items satisfied both IIC and IDV criteria overall, the Resilience (RE) and AU dimensions each showing more than one-third of their items with either low IIC or low IDV.

With regard to the uni-dimensionality of each dimension, 6 out of 8 dimensions showed acceptable INFIT values, while two items in each of the AU and *Relations with Family* (Rfa) dimensions, had INFIT values out of the acceptable range.

### Step 2: Cognitive debriefing see Additional file 1

Of the 21 persons included in step 2, nine completed the entire questionnaire, all interviewer administered. The other twelve participants stopped between questions 19 and 31 (out of a total of 41 questions) saying that the subjects being broached were too upsetting. All of these questions involved issues family life, friends and emotional well-being. Thematic analysis revealed two main reasons for interrupting participation: difficulty concentrating (*n* = 10) and fatigue caused by negative emotions experienced when reading the items. (*n* = 12). As one respondent pointed out: “*These questions are like a slap in the face*”. The questions considered to be most relevant to the concept of *quality of life* were those that dealt with self-confidence, self-esteem and the liberty to act and make one’s own decisions (5 respondents). Overall, only 4 subjects found the questionnaire acceptable: “*It shows that I am able to think, that I am not crazy, and that is reassuring”*. “*It's interesting to be brought up to date, to see whether I have changed my opinions*”.

*Open-ended* concerning the relevance and acceptability of the content of the S-QoL were identified.

#### Emotional difficulties induced by questions about family or friends

All items concerning family issues provoked unease in the conversation, and brought out negative feelings of sadness and nostalgia. Several subjects reacted in a hostile manner: “*I don't want to answer that question*”, “*I don't want to say any more in this interview*”, “*My family knows nothing about what's going on with me; it's upsetting to talk about it*”. One person thought that certain questions should not have been asked, because “*They are emotionally difficult*”,“*These questions hurt*”, “*They are a slap in the face for people who live on the street*”.

Concerning the family-related questions, another person said “*It's important, but it's private*”. Two interviewees suggested that the number of questions relating to family issues should be reduced.

#### Difficulty understanding

For 16 people, the instructions for scoring each item (*more than expected, less than expected*) were difficult to understand. They had to ask the interviewer for help.

In spite of the fact that all participants had an adequate level of French, some had trouble reading or understanding what they were reading: “*It's too difficult for me. Not so much, not so much..!.*”. Others had difficulty concentrating: '*I am too tired to read.’*

Regarding vocabulary, some participants found certain questions or terms difficult or impossible to understand.

#### Inappropriateness of certain items in the context of participants’ personal lives

Concerning the item 12 “I make efforts to work”, several respondents – most often those who had been unemployed for several years did not understand whether the question concerned their motivation to look for a job or their efforts to keep a job if they found one.

#### Areas of quality of life that were not explored

A specific problem was raised by four individuals who had difficulty responding to a questionnaire that was not their mother tongue: “*Most foreigners, when you ask them something, the answers have to be simple. Yes or no answers according to the mental state of the person. Sometimes nuances are impossible*”. In addition, although this was not a direct response to specific questionnaire items, some recent immigrants reported quality of life issues with regard to difficulty putting their emotions into words and making themselves understood in social interactions (Table 3).

**Table 3:** Areas not explored by the S-Qol Questionnaire (*N* = 21)

Area not explored	Items	
Life on the street	Home	*n* = 6
	Food	*n* = 5
	Identity Papers	*n* = 4
	Personal Safety	*n* = 2
Well-being	Freedom	*n* = 5
	Respect	*n* = 3
	Holidays	*n* = 2
	Hope	*n* = 2
	Hygiene	*n* = 2
Being a foreigner	Expressing one’sfeelings	*n* = 3

#### Definition of quality of life

A primary aspect of quality of life which six of the 21 participants spontaneously insisted upon was that of being able to satisfy their basic needs. A further 5 people underlined the need for normalcy, for having a life like other people: “*being like everyone else with family, children, work, sport, holidays, it's 50% of quality of life…, “A healthy lifestyle, not having to steal, to lie, to deal or sell drugs…*”. A third domain (4 participants) concerned freedom of action: “…*the questions of self-confidence, life, the liberty to do what you want”*; “*Quality of life is being able to travel, to come back, to stay, to leave again…*”.

A fourth area (4 people) was that of a positive relationship between oneself and others: “*No violence, no anger; love your neighbour and love life*”, “*I need to trust others more and have more faith in others, in life, in everything*”.

## Discussion

In Step 1 of the present study, a strong ceiling effect was found in the *Psychological Well-being* dimension (PsW), whereas the ceiling effect was low in the validation sample in the original S-QoL study [13]. This strong effect for the PsW dimension in the present study may be explained by the fact that the questionnaire was administered by an investigator working within partnership with the local outreach team. On the one hand, revealing psychological distress to a professional caregiver could bring them to be more supportive. On the other hand, over-estimation of psychological well-being may be linked to a bias related to social desirability [25].

Finally, a recent study by team that initially created the S-QoL scale showed that quality of life was better among individuals with deficits in metacognition and insight than in those without such deficits [26]. Subjects in the current sample were especially at risk of having deficits in metacognition and insight, linked to their many years of deprivation and hardship living on the street, the severity of their schizophrenia and associated addictions.

Overall, internal consistency reliability of the S-QoL was satisfactory except for the sentimental life dimension (SL alpha = 0.54), even if, by definition, the fact that this dimension includes only two items decreases the alpha value. The internal consistency of the AU dimension could be improved by deleting item 13, *'I can go out (to the cinema, for a walk, to a restaurant etc.’),* that interviewees reported as being inappropriate for someone living on the street.

Construct validity of the S-QoL remained acceptable regarding both unidimensionality and item-dimension score correlations, despite some limitations found for the RFa, RE and AU dimensions.

Regarding interviewer administration, the fact that many subjects preferred this form is to be noted. It introduces an interpersonal element that may influence subjects’ responses. The S-QoL questionnaire has been validated only in its self-administered version. Thus further research is needed to assess the influence, if any, of interviewer administration of the instrument, as well as addressing inter-rater reliability.

Areas of inadequacy identified in the S-QoL questionnaire during the second phase of the study include intrusiveness and lack of appropriateness subjects’ current living conditions; comprehensibility of certain questions; language and migration.

### Questions that are too intrusive

Certain questions, especially those concerning family issues, were experienced as being too intrusive, bringing up negative reactions and provoking rumination. This may be related to the frequency, in this population group, of dysfunctional families [16] and of childhood trauma [27]. The number of questions concerning subjects’ families should be reduced, and their phrasing should be adjusted to take into account the specificities of homeless populations, thus allowing more targeted data to be collected. For example: “*I am in contact with my family*”, “*I feel connected to my family*', rather than “*I speak with my family*”, “*My family listens to me*”, “*My family understands me*”.

### Comprehensibility issues

The phrasing of certain items and the vocabulary used should be adapted to this specific population. Evaluating items using *“less than expected”* and *“more than expected”* needs further debate.

### Relevance to subjects’ living conditions

Items concerning hobbies and work should be reworded to be socially inclusive of people living on the streets. In addition, items regarding access to basic needs such as safety, food and shelter should be phrased specifically to take into account subjects’ experience of living on the street. Although having a stable residence has been shown to be crucial for improving one’s quality of life [28], none of the items in the S-QoL touch on this question.

Thirdly, the question of the need for normality and of having self-confidence and trust in others may be explained by repeated experiences of being socially excluded and victimized, which may be connected to being identified as 'mentally ill', especially on the streets [29].

Fourthly, the question of liberty of action may be related to the question of being able to realize one's full potential when living with schizophrenia [30].

### Mother tongue and migration

It is important to take into account difficulties in social interaction experienced by migrants or by people answering a questionnaire that is not written in their native tongue. This is especially the case in France, where a significant number of mentally ill homeless people in France are foreigners [31] and where the incidence of schizophrenia is higher among recent immigrants than in the general population [32].

#### Limitations of the study

One limitation of the present study is its small sample size. However, overall results are comparable in terms of psychometric characteristics to those of a recent study conducted with the shortened version of the S-QoL (18 items versus 41) on a larger sample (*N* = 236) with similar characteristics, with the exception of weaker ceiling effects observed in the present study [33]. Moreover, to our knowledge, the present study is the first to explore the adaptability of the S-QoL (content validity, acceptability, appropriateness of self-administration) associated with a qualitative approach to a sample of homeless people living with schizophrenia. In addition, episodes of decompensation and cognitive deficits observed in people with severe schizophrenia are more frequent among those who have been homeless for long periods. This will inevitably influence quality of life assessment [26].

## Conclusions

The encouraging results in the present study concerning the reliability and internal validity of the S-Qol open up at least two areas for further research with regard to adapting the instrument for socially excluded groups. Firstly, certain areas of quality of life explored in the S-QoL awaken painful memories (social failure, dysfunctional families) and raise the question of the context in which this information is to be collected. After the questionnaire is administered, time should be allotted for discussion in order to address the possibility that responding to the S-QoL may have had a nocebo effect on subjects, a phenomenon which has been documented recently [34]. Secondly, this study emphasises the fact that less intrusive questions, more comprehensible vocabulary, more relevance to subjects’ living conditions and to their own definitions of priorities with regard to quality of life are needed to improve the S-QoL questionnaire.

Possible directions for future research include developing modular questionnaires with context-specific sections or creating quality of life instruments specific to socially excluded populations.

## Additional file

Additional file 1: Qualitative analysis of acceptability and content of the questionnaire. (DOCX 17 kb)

## Abbreviations

AU: Autonomy dimension of the S-QoL
CGI: Clinical Global Impression
IDV: Item discriminant validity
IIC: Item internal consistency
IRT: Item Response Theory
PhW: Physical well-being dimension of the S-QoL
PsW: Well-being dimension of the S-QoL
RE: Resilience dimension of the S-QoL
RFa: Relations with the family dimension of the S-QoL
Rfr: Relations with friends dimension of the S-QoL
RSM: The Rasch Rating Scale Model
SE: Self-esteem dimension of the S-QoL
SL: Sentimental life dimension of the S-QoL
S-QoL: Schizophrenia Quality of Life Scale

## Footnotes

1 European concensus conférence on homelessness: Policy recommandation of the jury. URL: http://www.feantsa.org/download/2011_02_16_final_consensus_conference_jury_recommendations_en-22191246727835177211.pdf?force=true
