# Supplementary material for: Relevance of a subjective quality of life questionnaire for long-term homeless persons with schizophrenia
Source: BMC Psychiatry. 2017 Feb 17;17:72. doi: 10.1186/s12888-017-1227-0 (PMC5314634; doi:10.1186/s12888-017-1227-0)
Supplement: Additional file 1: — Qualitative analysis of acceptability and content of the questionnaire. (DOCX 17 kb) [file 12888_2017_1227_MOESM1_ESM.docx]

Table 4. Qualitative Analysis of Acceptability and Content of the S-QoL Questionnaire

*( **^[[1]](#footnote-1)^*1** =emotional difficulties, **2** =understandability **3** =inappropriate for current living situation)

| **Item of the S-Qol 41** | **Problems** | | | **Propositions** |  |
| --- | --- | --- | --- | --- | --- |
|  | **1**  **n=11** | **2**  **n=16** | **3**  **N=14** |  | |
| 1- I’m confident in life |  |  | 1 |  | |
| 2-I fight to succeed in my life |  |  | 4 |  | |
| 3-I’m able to plan for my professional or personal future |  |  | 4 |  | |
| 4-I’m able to achieve my professional or personal projects |  |  | 4 |  | |
| 5-I feel self confident |  |  | 1 |  | |
| 6-I ‘m happy |  |  | 1 |  | |
| 7-I feel in a good mood, I a mat ease with myself |  |  |  |  | |
| 8-I feel in full bloom |  | 3 | 2 | *I have a fulfilling life* | |
| 9-I feel free to take decisions |  |  |  |  | |
| 10-I feel free to act |  |  | 2 |  | |
| 11- I have an active life |  |  | 4 | *I have a productive and fulfilling life* | |
| 12-I make efforts to work |  |  | 4 | *I work hard look for work* | |
| 13-I’m able to go out (cinema,  walks, restaurant . . .) |  |  | 8 | *I have hobbies* | |
| 14-I’m able to achieve my family and  sentimental projects | 11 |  |  |  | |
| 15-I’m in good physical shape |  |  |  |  | |
| 16-I’m full of energy |  |  | 1 |  | |
| 17-I do sports, I practise physical activities |  |  | 1 |  | |
| 18-I have a stable well balanced life style |  |  | 3 |  | |
| 19-I’m able to talk with my family | 11 |  |  |  | |
| 20-I’m helped, supported by my family | 11 |  |  |  | |
| 21-I’m understood by my family | 11 |  |  |  | |
| 22_I’m self-sufficient, independent of my  family | 11 |  |  |  | |
| 23-I see, meet my family | 11 |  |  |  | |
| 24-My family pays attention to me | 11 |  |  |  | |
| 25-I see, invite my friends or my relatives | 11 |  |  |  | |
| 26-I’m able to confide in someone |  |  |  |  | |
| 27-I’m helped, supported by my friends  or my relatives | 11 |  |  |  | |
| 28-I’m understood by my friends or my  relatives | 11 |  |  |  | |
| 29-I have friends | + |  |  |  | |
| 30-I’m satisfied with my love life | + |  |  |  | |
| 31-I feel comfortable when in public |  |  |  |  | |
| 32-I fear for my future |  |  |  |  | |
| 33-I feel useless |  |  |  |  | |
| 34-I feel anxious |  |  |  |  | |
| 35-I feel lonely |  |  |  |  | |
| 36-I have difficulty concentrating, thinking  straight |  |  |  |  | |
| 37-I get bored |  |  |  |  | |
| 38-I feel myself cut-off from the outside world |  |  |  |  | |
| 39-I fear accomplishing administrative  procedures |  | 6 |  | I fear doing paper work | |
| 40- I have difficulty expressing my feelings |  |  |  |  | |
| 41 -I have difficulty paying attention things to  my surroundings |  |  |  |  | |

1. [↑](#footnote-ref-1)
